# Optimising the value of the evidence generated in implementation science: the use of ontologies to address the challenges

**DOI:** 10.1186/s13012-017-0660-2

**Published:** 2017-11-14

**Authors:** Susan Michie, Marie Johnston

**Affiliations:** 1 Centre for Behaviour Change, University College London, 1-19 Torrington Place, London, WC1E 7HB UK; 2 Aberdeen Health Psychology Group, University of Aberdeen, Aberdeen, UK

## Abstract

Implementing research findings into healthcare practice and policy is a complex process occurring in diverse contexts; it invariably depends on changing human behaviour in many parts of an intricate implementation system. Questions asked with the aim of improving implementation are multifarious variants of ‘What works, compared with what, how well, with what exposure, with what behaviours (for how long), for whom, in what setting and why?’. Relevant evidence is being published at a high rate, but its quantity, complexity and lack of shared terminologies present challenges. The achievement of efficient, effective and timely synthesis of evidence is facilitated by using ‘ontologies’ to systematically structure and organise the evidence about constructs and their relationships, using a controlled, well-defined vocabulary.

## Background

Implementing research evidence into practice depends on changing human behaviour—at individual, organisational, community and population levels [1]. The pathways of implementation are generally complex, weaving in non-linear fashions through multi-level systems; but at every level, practice only changes when people do things differently. People may be managers, service commissioners and providers, ancillary, administrative and technical staff, policy-makers, politicians amongst others.

A considerable body of evidence about behaviour change in relation to health has accumulated, as evidenced by systematic reviews published in the Cochrane Library (Cochrane Effective Practice and Organisation of Care Group (EPOC) [2]). This evidence can be drawn upon to inform implementation. Frameworks abound, with a cross-disciplinary review identifying 83 theories [3, 4]. Many have been applied to implementation behaviours, mainly healthcare professionals’ behaviours such as clinical assessments and procedures, prescribing, advising and test ordering [5]. However, despite some successes, interventions to change behaviour have had modest and variable success. This to some extent reflects the large heterogeneity in interventions resulting in part from their complexity, with many interacting components, both the techniques themselves and their delivery features (e.g. mode, source and dose). In addition, contexts are heterogeneous with huge variation in intervention setting and target population, and context may influence the effectiveness of interventions. Although the majority of implementation and behavioural intervention research is conducted in the USA, the majority of global health challenges concern settings and populations in very different countries, cultures and contexts. A further source of heterogeneity is the variety in study methods used in intervention evaluations.

To make significant improvements, we want to be able to answer with confidence, variants of the questions posed by implementation and other researchers, policy makers and practitioners: ‘What works, compared with what, how well, with what exposure, with what behaviours (for how long), for whom, in what setting and why?’ and we want to be able to do this for questions that often lack large amounts of high-quality evidence. This will require very extensive searches to identify evidence and the ability to make inferences where evidence is lacking. Evidence of which behaviour change interventions are effective is required by a wide range of sciences (including implementation, behavioural, psychological, clinical, human factors and organisational sciences) and by those involved in developing policy and implementing interventions in practice.

## Limitations of current efforts to generate and apply evidence about behaviour change

Current efforts to systematically generate and apply the available evidence about behaviour change are limited by the quantity of evidence, its complexity and the lack of standardised terminology and structures for reporting interventions and their evaluations.

There is no shortage of publications on behaviour change and interventions. For example, Google Scholar searches identified over 145,000 publications for ‘behaviour change intervention’ in 2016 which reflects the high publication rate for the last 20 years. At this pace, only a very efficient system could aggregate the evidence and we currently lack such a system. Our limited search and review methods may additionally contribute to the problem of reproducibility of results as cognitive biases may influence our search terms and interpretation of findings [6]. As a result, it is difficult to ascertain what we know and where there are gaps in the evidence base. Even where good evidence exists and is synthesised, the resulting review and recommendations are inevitably out of date by the time it is complete, due to the laborious work involved [7].

The issue of quantity of evidence is exacerbated by its complexity. An effective aggregation of evidence should take account of the active content and delivery of the interventions, the theoretical basis for the intervention, the characteristics of populations and settings in addition to specifying the target behaviours and the methods of investigation. However, while evidence continues to be generated at a fast pace, there is no agreed framework for accumulating that evidence and as a result, evidence is fragmented and gaps in the evidence are difficult to identify.

Evidence synthesis is further hampered by the wide variability and quality of research methods used to evaluate the effectiveness of interventions. Even for the same behaviour, outcomes may be assessed in different ways, e.g. objective versus self-report measures, with different follow-up periods. Even when a randomised controlled trial design is used, interpretation of findings can depend on the support or treatment given to the control groups.

Complexity is exacerbated when reporting of interventions and studies is incomplete [8], a problem that is greater for behavioural than other non-pharmacological trials [9]. As a result, attempts to advance the science of behaviour change are hampered and effective interventions cannot be implemented reliably in practice. This means that there continues to be wastage of evidence due to the inadequacy of our terminology and structures for reporting behaviour change and interventions.

In sum, there is currently too much, incompletely reported evidence on behaviour change interventions for current methods to synthesise within an acceptable time frame. In addition, the systems available for aggregation of the evidence reflect a complexity requiring more human and funding resources than are available. Although these points have been made about behaviour change interventions, they apply also to implementation interventions.

## The potential of ‘Ontologies’ to advance collaborative and cumulative science

Recent advances in behavioural, computing and information sciences provide a step change in the possibilities for addressing complex questions efficiently, effectively and in a timely manner. Developments in information science include automating parts of the process such as the evaluation of risk of bias in trials. Methods of making synthesised evidence accessible are advancing but depend on success in generating useful search terms for accessing the accumulated evidence. In computer science, developments such as natural language processing, machine learning and artificial intelligence provide new opportunities for synthesising the burgeoning and fragmented scientific literature and making inferences where gaps exist [10, 11]. Computing systems have enabled more efficient identification of published reports of interventions [12] reducing the human load and increasing the speed of evidence synthesis. In addition, they offer the potential to integrate the evidence more effectively [13] and, in the long term, provide novel insights by identifying patterns and associations not detectable by current methods.

However, a review of automated evidence extraction methods noted the incompleteness of methods, pointing to the importance of having a strong framework or ‘ontology’ for organising the information that might be extracted [14]. An ontology is a systematic structure for organising knowledge about (1) entities (constructs) using a controlled vocabulary for labels and definitions and (2) their inter-relationships. They facilitate codification of this knowledge in a computer-readable format to enable organisation, reuse, integration and analysis [15]. In addition, the systematic organisation of knowledge by ontologies may lead to the generation of additional knowledge which is present but undetected in the evidence base as computers can investigate patterns in the evidence more rapidly and without the preconceptions of humans.

Developments in behavioural science are providing shared terminology and structures for conceptualising behaviour change interventions providing a method for more co-ordinated extraction and synthesis of evidence. Ninety-three behaviour change techniques, the active ingredients of behaviour change interventions, had been defined by 2013 [16, 17] organised hierarchically and shown to be used reliably; and others have since been identified. Meanwhile, statistical methods of combining techniques to explain outcomes are being advanced and initial typologies of context have been developed in relation to interventions [18], behaviours [19] and implementation [20]. More recently, a hierarchical taxonomy of modes of delivery based on more than 60 unique modes in use in published literatures has been developed [21].

Structures for conceptualising behaviour change evaluations and interventions have been developed such as Cochrane’s PICO ontology (specifying the population/problem, intervention, controls and outcomes) [22], and TIDieR (Template for Intervention Description and Replication) template for reporting the essential components of interventions offers consensus methods for organising and thus simplifying complexity [8]. The use of ontologies goes some way to capitalise on recent developments in order to address many of the problems raised above.

Behavioural science and implementation science could gain enormously by drawing on computational methods to optimise the value of current evidence. However, in order to make these gains, we need to make our evidence computer readable. This will require a consensus-based system of organising our knowledge that identifies the key ‘entities’ which define our evidence and which can be reliably recognised, on a limited scale, by humans. In sum, a behaviour change intervention ontology is required to allow accumulated evidence to be computer readable as a basis for more rapid and effective evidence synthesis.

## Implications for implementation science

If implementation science continues to depend on human integration of evidence using heterogeneous terminologies, we will accumulate more systematic reviews but is unlikely to achieve the theoretical understanding about behaviour change that is needed by implementation researchers and practitioners, and the research, policy and practice communities more broadly.

The Human Behaviour Change Project, funded by the Wellcome Trust from 2016 to 2020, aims to build on the scientific developments to provide a step change in our ability to synthesise evidence at scale, speed and granularity [23, 24]. It will, using artificial intelligence and machine learning, develop and evaluate a ‘knowledge system’ that continually scans the world literature on behaviour change interventions, automatically extracts key information, synthesises and interprets findings to generate new insights about behaviour change and improve prediction of intervention effectiveness.

The project will be collaborative throughout, generating data that can be used by the research community to address a wide range of research questions and involving a large international consortium of stakeholders and scientific advisors. The project will also build a user interface to allow researchers to access up-to-date syntheses and interpretations of evidence.

We believe that ‘business as usual’ is not an option if we are to make the step changes in evidence synthesis and in our capacity to answer the ‘big’ shared question for implementation and behavioural science: *What works, compared with what, how well, with what exposure, with what behaviours (for how long), for whom, in what settings and why?* Ontologies offer a way forward by enabling us to structure our knowledge to make it recognisable to computers in order to gain the scale and completeness required.

## Implications for research agenda

This work is in its infancy and suggests a wide range of research questions, a few of which we set out here:

1. Are there important aspects of implementation interventions which we cannot currently report and which (a) would benefit from the development of an ontology or (b) where an ontology is likely to be impossible?
2. How acceptable and feasible are taxonomies and ontologies to those conducting, reporting, interpreting, synthesising and implementing evidence?
3. Do taxonomies and ontologies lead to evidence reports which
  - Are more easily understood?
  - Are interpreted accurately, i.e. as in the original intervention? and
  - Lead to faster, more precise implementation of effective interventions?
